# Evaluating the promise of inclusion of African ancestry populations in genomics

**DOI:** 10.1038/s41525-019-0111-x

**Published:** 2020-02-25

**Authors:** Amy R. Bentley, Shawneequa L. Callier, Charles N. Rotimi

**Affiliations:** 1 Center for Research on Genomics and Global Health, National Human Genome Research Institute, National Institutes of Health, Bethesda, MD USA; 2 Department of Clinical Research and Leadership, The George Washington University School of Medicine and Health Sciences, Washington, DC USA

**Keywords:** Personalized medicine, Genome-wide association studies

## Abstract

The lack of representation of diverse ancestral backgrounds in genomic research is well-known, and the resultant scientific and ethical limitations are becoming increasingly appreciated. The paucity of data on individuals with African ancestry is especially noteworthy as Africa is the birthplace of modern humans and harbors the greatest genetic diversity. It is expected that greater representation of those with African ancestry in genomic research will bring novel insights into human biology, and lead to improvements in clinical care and improved understanding of health disparities. Now that major efforts have been undertaken to address this failing, is there evidence of these anticipated advances? Here, we evaluate the promise of including diverse individuals in genomic research in the context of recent literature on individuals of African ancestry. In addition, we discuss progress and achievements on related technological challenges and diversity among scientists conducting genomic research.

## Introduction

The genomic research landscape has transformed dramatically since the completion of the human genome project in 2003, but focus in the field has been constrained, in terms of worldwide populations included in this research. Representation in genomics studies has been dominated by populations descending from Europe. The concentration of research and resources within one ancestral group has the potential to further perpetuate and even exacerbate known disparities that negatively affect populations with ancestry that is underrepresented in research (see Box 1: “What do we mean by ancestry?”). There has been increasing recognition of the scientific and equity imperatives to increase the representation of diverse populations in genomic research,^1–9^ and initiatives are underway to address past shortcomings. As these initiatives are beginning to yield results and more findings from studies including diverse populations are accumulating, it is worthwhile to revisit the promises of this inclusion to evaluate whether the anticipated advances are, indeed, emerging. With a focus on the African continent, consistent with the authors’ expertise, we evaluate the evidence to support the expected progress from including diverse individuals in genomic research relative to: novel insights into human biology, improvements in clinical care, and enhanced understanding of health disparities (Table 1). The call for increased diversity in genomic research has been accompanied by the need for overcoming related technological challenges and for greater diversity among those conducting research. We also comment on the progress and achievements on these issues.

**Table 1:** Progress and challenges for achieving scientific promise of diversity and inclusion in genomics.

Area	Example of recent success	Remaining challenges
Insights into human biology	New loci and new variants within known loci in large projects including African ancestry individuals	Number of included individuals remains relatively low Even with increasing numbers of diverse individuals, preferential analysis and reporting of EUR results, which have achieved even larger sample sizes Because of the diversity among AFR populations and the reduced linkage disequilibrium across the genome, studies of AFR individuals will require even greater sample sizes than EUR to properly interrogate variation present
Improvements in clinical care	Identification of new pharmacogenomic loci relevant for individuals with African ancestry through consortia and new tools	Continued underrepresentation of non-European ancestry individuals in clinically important databases limits diagnostic accuracy for these individuals Need for more pharmacogenomic research in diverse populations Need for more data from which to derive polygenic risk scores specific to ancestry groups
Improved understanding of health disparities	Identification of genetic variation that may be contributing to “racial” differences in disease-relevant traits	Identifying appropriate biomarkers, genetic variants, and/or environmental factors for distinguishing risk instead of race categories
Infrastructure to include diverse voices	Infrastructure development within H3Africa to support the full participation of African scientists in high-level genomic research	Sustainability of current initiatives will require further commitment on the part of funding agencies and governments
Overcoming technological challenges	Development of a genotyping array for improved interrogation of genetic variation present in populations with primarily African ancestry	Analysis of populations of diverse and admixed ancestral backgrounds may require the development of novel techniques
Development of ethical guidelines	Development of ethical guidelines and training modules related to conducting genomic research through the H3Africa initiative	Sustaining mature and informed institutional review boards and ethics committees to address ethical issues that arise with the expansion of genomic research in Africa will require continued efforts

For clarity, and to be consistent with recently published guidelines for characterizing ancestry in genomic research,^10^ we here use “individuals of African ancestry” to describe sub-Saharan Africans and those who identify as African American or Afro-Caribbean. Similarly, we use “European ancestry individuals” to describe those who identify as “white” from Europe or America. However, the concepts presented may extend directly to all individuals with any degree of a particular ancestry, as ancestry at a genomic locus of interest may have important health effects, regardless of self-identification.

Box 1 What do we mean by ancestry? In the context of this paper, ancestry is defined using genetic variants based on the distribution of those variants in worldwide populations. An individual’s genome is a mosaic of segments from different ancestral populations. By comparing segments of DNA with the distribution of genetic variants in worldwide populations, it is possible to determine the likely “parental” or source population for each segment of DNA, indicating a component of the individual’s overall ancestry. Using this process to interrogate an individual’s entire genome, the proportion of an individual’s genomic inheritance from specific ancestral populations can be estimated. Importantly, someone’s genetic ancestry may have little to do with their identity in terms of race or culture. An individual with a relatively small proportion of African ancestry may not self-identify as “Black” or “African American”, yet they may have African ancestry at a specific region of the genome where that ancestry may confer risk, for instance, by carrying a pharmacogenomic risk allele that is more prevalent among those with African ancestry. Based on our most recent analysis of 282 global samples, there are at least 21 ancestries.^139^ Notably, mixed ancestry is the norm across samples and continents, and the vast majority of individuals (an estimated 97.3%) show ancestral heterogeneity.^139^ Finally, individuals’ ancestry estimates may change over time as our reference datasets become more diverse.

## Insights into human biology

A substantial proportion of human genomic variation was left behind during the out-of-Africa migration.^11^ Therefore, insights into the implications of this variation can only come from research conducted in those of African ancestry. This reservoir of relatively untested African sequence variation has fostered high hopes regarding the potential for novel findings when better representation in genomic research was achieved.^6^ Notably, expected advances based on findings in those of African ancestry are not limited to those of that ancestry, as can be seen with the discovery of variants in *PCSK9* that dramatically reduced low-density lipoprotein cholesterol concentrations. Although these variants were discovered in samples from African Americans, the benefit of drugs targeting *PCKS9* extends beyond ancestry-defined subgroups.^12,13^ In general, common complex diseases require the aggregation of large datasets to yield results. Achieving the needed datasets of participants with African ancestry has been a challenge due to the relatively limited numbers and sample sizes of cohorts from diverse populations. Now that studies of African ancestry individuals are increasing in sample size, are we seeing the expected novel results? A recent evaluation of data included in the NHGRI-EBI GWAS Catalog, which is the most complete record of published genome-wide association studies, found that only 2.4% of the individuals included in the catalog were of African ancestry.^10^ Even at such a relatively small proportion, the promise of new findings with greater inclusion is clear: they contributed a larger than expected proportion of associations in the catalog (7%).^10^

**Fig. 1:**
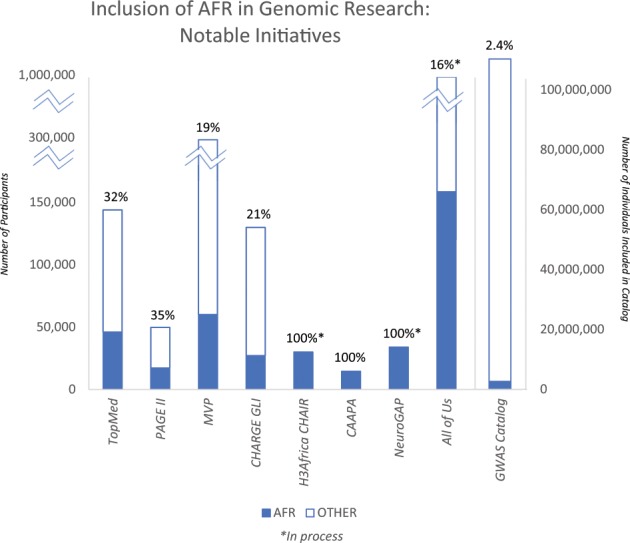
Inclusion of AFR in genomic research: notable initiatives. Data for figures taken from the following sources: TOPMed,^106^ PAGE II,^107^ Million Veteran Program (MVP),^108^ CHARGE Gene–Lifestyle Interactions (GLI),^109^ Cardiovascular H3Africa Innovation Resource (CHAIR),^35^ CAAPA,^110^ NeuroGAP-Psychosis,^36^ All of Us,^23^ and the GWAS Catalog^10^.

New initiatives and large consortia with a focus on increasing the representation of diverse populations in research, such as TopMED and PAGE II, are beginning to bear fruit (Fig. 1; Table 2). In addition to these large-scale efforts, the inclusion of individuals with African ancestry in other studies has led to the identification of novel loci in recent studies of obesity,^14^ type 2 diabetes,^15,16^ metabolic syndrome,^17^ skin pigmentation,^18^ suicide,^19^ multiple sclerosis,^20^ cleft palate,^21^ and Epstein-Barr virus immune response.^22^ The National Institutes of Health’s recently launched All of Us Precision Medicine Initiative aims to recruit 1 million Americans, with a particular focus on representing the nation’s diversity (http://allofus.nih.gov).^23^ With estimates of early recruitment showing up to 75% from groups who are underrepresented in health research, this effort promises to yield a considerable number of participants of diverse ancestries.^24^ Although the development of large, multi-ethnic resources is a clear advance, diligence on the part of researchers, funders, and reviewers is required to ensure that these resources are used to their fullest extent. It is common to exclude non-European ancestry samples from analyses, despite the presence of a sufficient sample size for stratified analysis (such as in UK Biobank),^25^ or to limit analyses in these samples to replication or confirmation of findings from the analysis of European ancestry samples.

**Table 2:** Some insights into human biology from initiatives prioritizing inclusion of participants of African ancestry.

Initiative	Novel loci and novel associations within known loci^a^
Trans-Omics for Precision Medicine (TOPMed)	Serum lipids^111^ Lipoprotein(a)^112^ Chronic obstructive pulmonary disease^113^ Rh antigens^114^
Population Architecture using Genomics and Epidemiology II (PAGE II) study	Adiposity traits^115–117^ Serum lipids^118^ Glycemic traits^119^ QT interval^120^ Age of reproductive events among women^121^ C-reactive protein^122^ Blood pressure^123^ Complex traits^107^
The Million Veteran’s Program (MVP)	Serum lipids^124^ Blood pressure^108^
CHARGE Gene–Lifestyle Interactions^125^	Blood pressure^109,126,127^ Serum lipids^128–130^
The Consortium on Asthma among African-ancestry Populations in the Americas (CAAPA)	Understanding of genetic diversity among individuals with African ancestry^131,132^ asthma risk^133^
MalariaGEN	Severe malaria risk^134^ Cerebral malaria and alphathalassemia^135^ Malaria resistance^136^ G6PD and malaria risk^137^ Population genetics^138,139^

^a^Including novel loci for the studied trait as well as novel associations within known loci

The above projects largely reflect inclusion of African Americans in genomic research, but representation on the African continent, the birthplace of humanity and home to the greatest genomic diversity on the planet, has been more limited. In response to this need, the Wellcome Trust and the National Institutes of Health have supported African-led genomic research through the Human Heredity and Health in Africa (H3Africa) initiative.^26,27^ Now in its 6th year, H3Africa has received over 170 million USD and sponsored nearly 50 research projects.^28^ At the time of the writing of this manuscript, H3Africa had reached 54,000 research participants, and convened meetings and workshops with over 2000 attendees.^28^ This initiative has produced 382 trainees and 219 publications.^28^ While primary results from the H3Africa projects for the study of complex disease traits have not yet been published, they are poised to make substantial contributions to increasing diversity in genomic research given the following enrollment numbers: a study of over 10,000 individuals for cardiometabolic disease risk (AWI-GEN),^29,30^ a study of 6000 T2D cases and 6000 controls (H3Africa T2D Study),^31^ a study of kidney disease (*n* = 4000 cases and 4000 controls) (H3Africa Kidney Disease Research Network),^32^ a study of 3000 stroke cases and 3000 controls (SIREN),^33^ a study of over 11,000 women on Human Papilloma Virus and cervical cancer risk (African Collaborative Center for Microbiome and Genomics Research),^34^ and a combined resource for cardiovascular studies with over 30,000 participants (CHAIR).^35^ Another noteworthy initiative is the Neuropsychiatric Genetics of African Populations-Psychosis (NeuroGAP-Psychosis) project, a study of the genetics of schizophrenia and bipolar disorder recruiting 34,000 participants from Ethiopia, Kenya, South Africa, and Uganda.^36^ These genomic studies and associated consortia will undoubtedly greatly enhance the detection of novel loci for complex disease risk.

## Improvements in clinical care

**Fig. 2:**
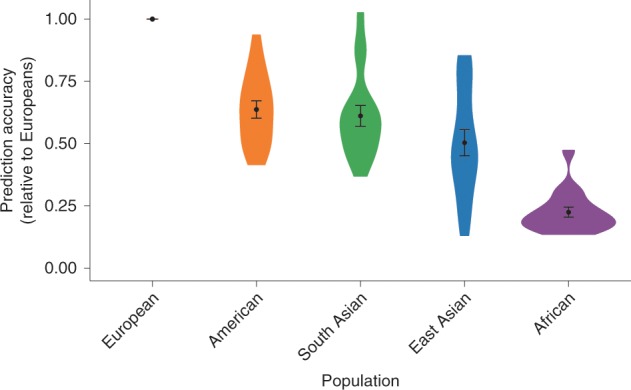
Prediction accuracy relative to European-ancestry individuals across 17 quantitative traits and 5 continental populations in the UKBB: All phenotypes shown here are quantitative anthropometric and blood-panel traits. Prediction target individuals do not overlap with the discovery cohort and are unrelated. Violin plots show distributions of relative prediction accuracies, points show mean values, and error bars show s.e.m. values. [Reprinted by permission from Springer Nature Customer Service Centre GmbH: Springer Nature, Nature Genetics, “Clinical use of current polygenic risk scores may exacerbate health disparities”, Alicia R. Martin et al.^42^].

A key motivation for conducting genomic research is to facilitate improvements in clinical care. A promising outcome of the success of genome-wide association studies (GWAS) of common diseases is the development of polygenic risk scores (PRS): scores that can be calculated for an individual based on the presence or absence of risk variants identified in large GWAS studies. The promise of these scores for clinical use is gaining increased attention.^37,38^ For instance, risk scores developed using known risk variants have been able to identify the 8.0% of the population with more than a three-fold increased risk of coronary artery disease.^39^ However, PRS depend on underlying genomic research, which has predominantly been conducted in European ancestry individuals. European ancestry individuals are overrepresented in PRS research at 460% of what it would be if representation matched the proportion of the worldwide population. In contrast, African ancestry individuals are underrepresented (only 17% of expected proportion based on worldwide population).^40^ As a result, these PRS represent only a subset of the global human population and have limited portability across ancestries.^1,41^ Some of this lack of portability reflects differences in characteristics of the genome that have accumulated as a result of migration patterns. For instance, as a result of genetic drift and selection pressures, the presence and frequency of alleles vary across ancestry groups.^11^ PRS can only capture the genetic associations observed in the population in which the underlying GWAS is conducted. An effect of variants that are not sufficiently frequent (or present) in that analysis may influence trait outcomes in a population of a different ancestry, yet the ancestry-unmatched PRS will not reflect those effects and the PRS will give less accurate results. Additionally, population history has led to a generally reduced linkage disequilibrium structure across the genome among those of African ancestry. Although an untested causal variant may be the same across populations, the variant which tags that effect may differ across ancestry groups. In this case, the risk associated with a particular variant may be misattributed and also lead to ancestry-unmatched PRS inaccuracies. The underperformance of the PRS when discovery and target populations are mismatched in ancestry has been demonstrated when PRS constructed based on GWAS findings in European ancestry individuals were tested in those of African ancestry. In UK Biobank data, prediction accuracy was 4.5-fold lower in African ancestry samples compared to European ancestry samples^42^ (Fig. 2). Similarly, the median effect size for PRS for a variety of traits in African ancestry samples was only 42% of that in matched European ancestry samples in an analysis of published PRS studies.^40^ In contrast, risk scores based on data derived in African Americans have been demonstrated to perform significantly better in African Americans than risk scores based on studies predominantly including those of European ancestry.^43^ The PRS could be considered as a new disease biomarker to be evaluated alongside more traditional measures of risk. It is common that the distribution of disease biomarkers varies across ancestries, often leading to adjustments in biomarker calculations (for instance, predicted values for spirometry or estimated glomerular filtration rate). However, PRS uniformly performs more poorly in individuals with African ancestry (and in all understudied populations), and minor ancestry-specific adjustments, such as have been used with other biomarkers, will not be able to make up for this underperformance.^42^ The complexities of PRS in the context of ancestry and the potential to exacerbate health disparities has been addressed in-depth in recent articles.^40,42^ Importantly, in addition to limitations in transferability conferred by ancestry-related genomic differences, there can be substantial variation in the performance of PRS for complex genetic traits based on methodological decisions^40^ and on environmental context.^44^ If PRS do become useful clinically, the limitations of applying data from a subset of the global population to all global populations are clear. Disparities in clinical care will persist for those with non-European ancestry: medical technology will be less capable of identifying risks, targets, and potential interventions for the majority of patients globally that represent greater genomic heterogeneity. While advances in understanding and adjusting for biases when PRS are applied across ancestries is useful, real progress in PRS for those of African ancestry is tied to increasing the number of large GWAS of African ancestry individuals on which to base these PRS.

Another important area of genomic research in which increased diversity is vital for equitable clinical care is pharmacogenomics: identifying genetic variants that may influence an individual’s response to drugs. Depending on the pharmacogenomic variant carried by an individual, treatment outcomes could range from a lack of effect to potentially life-threatening adverse effects. Using genomic data to protect patients from harm or improve their treatment is a key motivation for precision medicine. As the genetic variants present in individuals may vary by their ancestry, limited pharmacogenomic research in diverse populations puts individuals with ancestry from an underrepresented group at more risk of an unanticipated drug response than those in better-studied populations. The African American Cardiovascular Pharmacogenetic Consortium (ACCOuNT) has been established to address this gap and aims to discover novel pharmacogenetic variants in African Americans and to incorporate these variants into clinical recommendations.^45^ Recent efforts focusing on those of African ancestry have identified novel loci that may help to explain the diminished response to inhaled corticosteroids in African Americans with asthma,^46–48^ though the low number of available studies with individuals of African ancestry to confirm findings was acknowledged as a continuing limitation.^47^ The first GWAS of neutrophil counts during clozapine treatment (for schizophrenia) conducted in individuals of African ancestry identified a key role for the well-known African ancestry-specific Duffy allele in risk of neutropenia upon treatment, thereby improving our understanding of the differential rates of discontinuation of clozapine by ethnicity.^49^ Major warfarin-associated bleeding occurs more commonly among those with African ancestry, though genetic risk factors associated with this serious outcome had not been well-studied in these individuals. A recent case-control study among individuals of African ancestry^50^ was able to identify a locus that associated with more than eight times greater risk of major warfarin-associated bleeding. Risk variants at this locus are relatively common among individuals with African ancestry (minor allele frequency = 0.05) but absent in populations without any African ancestry. Also, potentially relevant warfarin dosing haplotypes, common in Africans but rare in other ancestry populations, have not been systematically evaluated.^51,52^ Although pharmaceutical drugs can be prohibitively expensive in many African countries, the potential for pharmacogenomic discoveries to increase safety and effectiveness should not be overlooked. Some countries, for instance, consider research findings related to pharmacogenomic alleles that have ramifications for their countries prior to selecting available medicines for use locally. As the prevalence of a *CYP2D6* duplication that causes serious adverse outcomes with codeine use is 30% among Ethiopians, and broad genotyping is not feasible, the use of codeine has been banned in Ethiopia.^53^ It is important to note, however, that because of the great genetic diversity within African ancestry individuals, care must be taken not to group those of African ancestry into broad categories with the expectation of genetic similarity. For instance, HLA-B*5701 is associated with increased incidence of abacavir hypersensitivity, a life-threatening condition among HIV patients being treated with this drug. The frequency of this variant ranges from virtually absent among the Yoruba in Nigeria to 13.6% among the Masai in Kenya. Among the Luhya, also in Kenya, the variant is only present in 3.3% of individuals. In this case, neither “African” nor “Kenyan” are sufficiently precise to describe risk at this pharmacogenomic locus.^54^ While there are examples of recent progress, continued pharmacogenomics research is needed to form a clearer picture of the distribution of pharmacogenomics variants across diverse African populations.^3,54^

Inclusion and diversity are also of critical importance for the use of genomic information in clinical diagnosis. Translation of genomics to clinical practice depends upon what genomic studies have been conducted. Analyses of important databases for reporting genomic results illustrate the continuing underrepresentation of populations with significant ancestry outside of Europe: individuals of European ancestry constitute 55.8% of the observations in the Genome Aggregation Database (gnomAD);^2^ and the Genome-wide Association Study Catalog and the database of Genotypes and Phenotypes both have significantly fewer studies of African, Latin American, and Asian ancestral populations compared to European ancestry populations.^2–4^ This current reality highlights the comparatively reduced ability for clinicians to assess clinically significant variants in individuals with diverse ancestry, particularly when those variants are not shared across populations. Illustratively, genetic testing for cardiomyopathy in European ancestry individuals is more likely to positively identify pathogenic variants than testing conducted in underrepresented minorities in the US, while genetic testing in underrepresented minorities is more likely to be inconclusive.^5^ African ancestry individuals were also more likely to have variants that were misclassified as pathogenic for hypertrophic cardiomyopathy because of a lack of ancestry-matched controls for comparison.^55^

## Improved understanding of health disparities

Although the social and economic factors are the most significant contributors to health disparities, genomic research has the potential to help unravel disparities in health outcomes that are inappropriately attributed to race. The discovery of kidney disease risk variants in the *APOL1* gene that are found predominantly in individuals with African ancestry demonstrated this potential. These variants are thought to have risen in frequency among some African populations through selection pressure due to protection from Human African Trypanomiasis, yet these variants are associated with a marked increase in the risk of kidney diseases of varying etiologies.^56^ Well-known differences in kidney transplant outcomes that had been attributed to race were found to be better described by *APOL1* genotypes. For instance, kidney transplant survival is poorer when the donor is African American compared to European American, but after accounting for those African American donors with the high-risk genotype, African ancestry is no longer associated with such outcomes.^57–59^ While stories like that of *APOL1* and kidney disease are likely uncommon, other health disparities are being investigated for genomic contributions, fueled by the increasing availability of genomic data for diverse populations. A *G6PD* variant that is common among those with African ancestry (but not those without African ancestry) leads to decreased HbA1C levels, irrespective of blood glucose, which may lead to an estimated 2% of African Americans with T2D to remain unidentified by HbA1c screening.^60^ A separate study reported a role of genetic factors in the difference in distribution of HbA1c by ancestry, also highlighting an association between the variant for hemoglobin S (“sickle cell”) in this trait.^61^ Additional discoveries have been made for the genetic factors underlying differences in the frequency of certain cancers by ancestry. For instance, in a pan-cancer analysis,^62^ higher degrees of chromosomal instability were observed among African American (vs. European American) patients with breast, head and neck, and endometrial cancers, while less chromosomal instability was observed in African American patients with kidney cancers.^62^ A study of triple-negative breast cancer identified different mutation profiles in the tumors of African American vs. European American patients, informing the higher prevalence of this subtype of breast cancer observed among African Americans.^63^ A recent study of prostate cancer disparities identified a protective allele that rose to high frequency in Europe due to linkage with a locus for skin pigmentation under selection pressure.^64^ The distribution of D-dimer, a cardiovascular disease risk biomarker, is higher among African Americans than among individuals with only European ancestry, and some genetic factors have been identified that contribute to that difference.^65^ Outcomes with treatment for chronic infection with hepatitis C virus vary, with African Americans having lower rates of treatment response compared to other ethnic groups. The C allele of rs12979860, a variant near *IL28B*, was found to be associated with improved treatment response among African Americans, European Americans, and Hispanics, and the inter-ancestry differences in frequency of this allele appear to explain approximately half of the disparities in treatment response rate.^66^ This variant was also associated with spontaneous clearance of the hepatitis C virus, and worldwide allele frequencies suggest that it may have been under selection in human history.^67^ Fortunately, newer treatment options have increased response to ~90% of patients, regardless of ancestral background.^68^ With continuing work on understanding how genetic factors contribute to health disparities, inadequate characterizations based on race can be replaced by screening for relevant markers, with improved targeting and the potential for novel biological insights.

## Overcoming technological challenges

To efficiently capture the wide range of genomic diversity among African participants, genotyping tools must be optimized for interrogating African genomes. GWAS arrays developed using genetic information derived largely from populations of European ancestry are inefficient when used within the context of research involving non-European populations, particularly the diverse groups that comprise Africa. There have been several notable efforts to improve the genomic diversity represented in commercially available genotyping arrays, including the Multi-Ethnic Genotyping Array (MEGA) produced by Illumina in partnership with the PAGE and CAAPA Consortia^69^ and the Affymetrix Axiom PanAFR Array, with a focus on coverage of 1000 Genomes AFR populations.^70^ More recently, the H3Africa Consortium and Illumina have developed a GWAS array with 2.5 million markers based on whole genome-sequencing data from thousands of samples originating from African research participants.^3^ This genotyping array better represents and reflects the range of common variants in populations across the continent, facilitating the interrogation of variants that had not been previously evaluated as they were not captured by previous tools.

After collection of genome-wide genotype data, it is common practice to impute additional genotypes prior to analysis. Accurate outcomes for imputation are dependent on the selection of the appropriate reference panel, as imputation algorithms incorporate linkage disequilibrium patterns, which vary across ancestries. Due to the generally reduced linkage disequilibrium and greater genetic diversity among African ancestry individuals, imputation accuracy using a comparably sized reference panel is reduced for those of African vs. other ancestries.^71^ Additionally, the reference panels publicly available for imputing African ancestry samples have been limited, predominated by the 1000 Genomes Project data. The situation has improved, however, with additional reference panels that include African ancestry individuals. High performance and accuracy for imputation with African Americans was observed for reference panels from 1000 Genomes,^11^ the Haplotype Reference Consortium (HRC, which includes 1000 Genomes data and a large number of predominantly European ancestry samples^72^), and the Consortium on Asthma among African-Ancestry Populations in the Americas (CAAPA^73^).^74^ These reference panels are available at both of the commonly used publicly available imputation servers: the Sanger Imputation Service (1000 Genomes and HRC: https://www.sanger.ac.uk/science/tools/sanger-imputation-service) and the Michigan Imputation Server (1000 Genomes, HRC, and CAAPA: https://imputationserver.sph.umich.edu/index.html#!). Additionally, the Michigan Imputation Server is preparing to offer a reference panel using TOPMed data. The African Genome Resource (AGR), available through the Sanger Imputation Service, is currently the largest publicly available reference panel of African ancestry individuals, including all 1000 Genomes individuals and an additional ~2000 samples from Uganda and ~100 samples each from 5 populations in Ethiopia, Egypt, Namibia, and South Africa. Notably, imputation using the AGR and the 1000 Genomes reference panel was shown to achieve high quality and accuracy even in the presence of five-way admixture in a South African population.^75^ Additionally, it is anticipated that data from H3Africa will be made available as an imputation resource.^76^

As the price of sequencing technologies continue to decrease^77^ and bioinformatic advances reduce the time and expertise burdens associated with processing these data, it is possible that sequencing may become more common. Importantly, the ancestry biases present in GWAS arrays and imputation are not a concern when using sequencing technology.

## Infrastructure to include diverse voices

The predominance of European ancestry individuals is observed in those represented in genomic research as well as in the identities of those conducting research.^7,78–80^ In the US, for example, the proportion of those in the academic doctoral workforce (for science, engineering, and health) who are African American has only increased from 5.8% in 1997 to 8.9% in 2017,^78^ although African Americans represent 13.4% of the population.^81^ Unfortunately, this lack of diversity among genomic researchers leads to the loss of perspectives that are important in developing hypotheses as well as directing research among diverse individuals, and disparity in terms of research leadership and output among African scientists remains.^82^ Sustained genomic research capacity in any setting relies on engaged investigators and frameworks that can support the development of datasets relevant to the local population, infrastructure for computation and analysis, and expertise for the translation and implementation of genomics and bioinformatics information into clinical practice. African countries have historically been challenged by poor research infrastructure and limited training and professional opportunities.^27^ In appreciation of this obstacle faced by African researchers, and with an eye toward the elevation of researchers along with the increased inclusion of African samples, sustained capacity-building is a keystone of the H3Africa Initiative.^83^ NeuroGAP has also established the Global Initiative for Neuropsychiatric Genetics Education in Research (GINGER), which will support the training of early-career investigators from the countries in which research is being conducted (Ethiopia, Kenya, South Africa, and Uganda).^84^ The topics for training are determined through the participation of the fellows and their African mentors, so that their needs shape their experiences.^84^

H3Africa supports the development of large collaborations across multiple centers, leverages synergies between studies, and provides critical training to African researchers. The pan-African bioinformatics network, H3ABioNet, was designed to serve the consortium and assists with data quality control and analysis.^85^ In addition, it has advanced infrastructure and professional skill development through workshops and meetings and accredited enrichment programs to advance skills related to the conduct of genome-wide association studies and the analysis of next generation sequencing data. Investigators have learned how to conduct GWAS and analyze genome-sequencing data. H3ABioNet has also established infrastructure for computing at different institutions across the continent. Through its monitoring and training efforts, the network has provided bioinformaticians, researchers, medical professionals, systems administrators, and software developers with the tools to utilize and maintain the computing infrastructure.^86^

One of the ways that the emergence of the voices of African researchers may be observed is in the focus on diseases and traits of significance to Africa among new Africa-led work. For instance, H3Africa-funded projects have supported work on Human African Trypanomiasis^87–92^ and sickle cell disorders.^93–95^ H3Africa provides a model that could be useful to include diversity in leadership in other parts of the world, including in America.

## Development of ethical guidelines

As the African genomic research environment expands to examine genetic, environmental, and other contributions to health and diseases, new and familiar ethical questions and concerns will arise that warrant attention to African perspectives. Investigations that employ emerging research tools (e.g., mobile health applications, sensors, wearable devices), for instance, must consider concerns related to genomic research that incorporates behavioral and physiological data.^96^ For instance, an individual’s ability to consent may be complicated in some African cultures by expectations of involvement of family and community in decision-making,^97^ as has been observed in indigenous people of the Americas.^98^ Ethical guidelines for these types of scenarios may need to be reevaluated as genomic research capacity grows within African contexts and produces researchers, institutions, and findings that are more accessible locally. Importantly, African countries must have the opportunity to influence the policies for the design and dissemination of genomic research for collaboration to be sustainable. Concerns over parachute or safari research remain where African researchers are left out of the full research process, invited to collaborate only as is useful for sample collection, but without any benefit of the research returning to support African researchers or infrastructure. Community engagement is recognized as a critical strategy for addressing such challenges, understanding research priorities, respecting cultural concerns, and countering concerns of exploitation.^99^ Others have highlighted the importance of tailoring community engagement strategies to low resource settings, including members of disadvantaged groups in the engagement process, and structuring deliberations in ways that minimize power differentials in community settings.^100^ Currently there are collaborations between African and non-African researchers “rooted” with a firm foundation within Africa that emphasize capacity building and shared scientific contribution, such as the Viral Hemorrhagic Fever Consortium (www.vhfc.org) and the African Center of Excellence for Genomics of Infections Disease (acegid.org).^101^ H3Africa has the formation of equitable collaborations, African researcher-led science, capacity building, and infrastructure development among its central tenets.^76,83^ Capacity building on the continent addresses concerns related to exploitation and parachute research through concomitant ethics and governance mechanisms, such as those created by H3Africa. Additional questions related to benefit sharing, privacy, confidentiality, and group harm should also be examined with emphasis on concerns specific to African populations.^102,103^ To address these concerns, H3Africa has provided an excellent foundation by developing guidelines and ethics training modules for ethics committees on the continent that will assess ethical concerns.^104^

Given the importance of data sharing to genomic research, genotype and phenotype data from the H3Africa research studies are stored in the European Genome Phenome Archive (EGA) but access to data and biological samples is determined by an independent H3Africa Data and Biospecimen Access Committee (DBAC) comprised of mostly African members who consider the informed consent process and stipulations provided by the original ethics committees who approved the studies. Through collective action, members of H3Africa have developed ethical guidelines related to data sharing and processing, consent for the sharing and uses of African samples and data, and issues related to study coordination and the shipping of samples externally.^86^ Example guidelines include rules and embargos that are sensitive to local infrastructural challenges, thus according to African investigators relatively sufficient time to publish the findings of their primary research questions first.^26,105^ The training modules and guidelines provide a framework and foundation for existing and future ethics committee members. Strategies to ensure that ethics committee members remain engaged, well-informed, and committed to the committee will be critical to ongoing developments related to ethical guidance.

## Conclusion

The need for inclusion of individuals of diverse ancestral backgrounds and identities in genomic research has long been understood, both from the perspective of scientific necessity and equity. Recent initiatives have embraced this call for diversity, including H3Africa, TopMed, CAAPA, PAGE II, and the Million Veteran Program, with more projects, such as large-scale H3Africa projects and the All of Us study seeking to represent US diversity, underway. As results from these and other efforts are reaching the literature, it is warranted to evaluate whether the anticipated benefits of the inclusion of diverse populations in genomics are being realized. Indeed, the increased number of participants with African ancestry is facilitating the discovery of novel genomic loci and establishing new variation of interest within known genomic loci. Importantly, the focus on increasing the number of African ancestry individuals in research is occurring alongside efforts to increase capacity and supporting infrastructure for diverse researchers, and to do so equitably. Clinical and pharmacogenomic studies are improving the data available to accompany integrated social and environmental research on the underlying causes of health disparities. Despite these advances, attaining meaningful representation of diverse populations across the breadth of genomic research that is adequately powered to inform health disparities research will require sustained and continuing efforts.
